# Deep learning based emulator for predicting voltage behaviour in lithium ion batteries

**DOI:** 10.1038/s41598-024-80371-9

**Published:** 2024-11-21

**Authors:** Kanato Oka, Naoto Tanibata, Hayami Takeda, Masanobu Nakayama, Syuto Noguchi, Masayuki Karasuyama, Yoshiya Fujiwara, Takuhiro Miyuki

**Affiliations:** 1 Department of Advanced Ceramics, Nagoya Institute of Technology, Gokiso, Showa-ku, Nagoya, Aichi 466-8555 Japan; 2 Department of Computer Science, Nagoya Institute of Technology, Gokiso, Showa-ku, Nagoya, Aichi 466-8555 Japan; 3 Consortium for Lithium Ion Battery Technology and Evaluation Center (LIBTEC), 1-8-31 Midorigaoka, Ikeda, Osaka 563-8577 Japan

**Keywords:** Battery emulator, Long short-term memory (LSTM), Lithium-ion batteries (LIBs), Deep learning, Time series analysis, Charge–discharge behaviour, Cheminformatics, Electrochemistry

## Abstract

This study presents a data-driven battery emulator using long short-term memory deep learning models to predict the charge–discharge behaviour of lithium-ion batteries (LIBs). This study aimed to reduce the economic costs and time associated with the fabrication of large-scale automotive prototype batteries by emulating their performance using smaller laboratory-produced batteries. Two types of datasets were targeted: simulation data from the Dualfoil model and experimental data from liquid-based LIBs. These datasets were used to accurately predict the voltage profiles from the arbitrary inputs of various galvanostatic charge–discharge schedules. The results demonstrated high prediction accuracy, with the coefficient of determination scores reaching 0.98 and 0.97 for test datasets obtained from the simulation and experiments, respectively. The study also confirmed the significance of state-of-charge descriptors and inferred that a robust model performance could be achieved with as few as five charge–discharge training datasets. This study concludes that data-driven emulation using machine learning can significantly accelerate the battery development process, providing a powerful tool for reducing the time and economic costs associated with the production of large-scale prototype batteries.

## Introduction

In recent years, researchers have increasingly focused on rechargeable lithium-ion batteries (LIBs) to address energy and environmental issues^1,2^. Thus far, LIBs are used as power sources for portable devices such as mobile phones, laptops, and digital cameras^3^. Furthermore, over the past decade, the use of LIBs in electric vehicles (EVs)^4^ has attracted significant attention^5,6^ owing to their advantageous features, such as high energy density, low self-discharge, and long life. However, unlike power supplies for portable equipment, LIBs for EVs must deal with irregular charging/discharging processes or changes in the power output depending on the driving conditions. In the development process of EVs, test drives are conducted to consider various driving conditions, resulting in the battery experiencing a variety of electric current and voltage input–output changes corresponding to those conditions. However, the production of large-scale prototype batteries for EV test drives without access to mass production lines often incurs significant time and economic costs^7^. Instead, a battery emulator^8^, which is an electric power device that mimics the charge/discharge behaviour of batteries, can reduce the time and economic costs associated with the fabrication of prototype batteries and driving tests of EVs. Typical battery emulators^9^ operate on a digital platform^10^, providing power supply and control to connected devices through stationary equipment. Although emulators using physics-based models, such as multi-physics methods^11,12^, exist, inputting numerous parameters^13,14^ to reproduce the performance of experimental prototype batteries is required and is highly challenging. Moreover, the complexity of constructing multi-scale models for LIBs has been well-documented^15^, and the software used to obtain the simulation dataset for this study also required numerous parameters. As such, we attempted to build a data science–driven battery emulator using machine learning based on a time (*t*)–current (*I*)–voltage (*V*) dataset during battery charge–discharge cycles. Specifically, we aimed to reproduce the charge–discharge behaviour by training a time-series deep learning model with long short-term memory (LSTM) to predict the output voltage profile from arbitrary charge–discharge schedules (current control). In previous studies using time-series analysis, there have been reports on predicting the lifespan of LIBs^16^ and forecasting capacity degradation^17^. Zhang et al.^18^ determined the occurrence of LIB failures with high reliability and predicted the remaining useful life of LIBs using an LSTM model. Liu et al.^19^ proposed a model combining Gaussian process regression and LSTM to evaluate battery health, including the future effective capacity and the remaining service life of batteries. Other studies have combined time-series deep learning models with convolutional deep learning methods^20,21^. Time-series analysis has been widely used for predicting the degradation and state of charge (SOC) of LIBs. Therefore, the time-series deep learning method is suitable for data-driven battery emulators.

In previous studies on the time-series machine learning prediction of battery lifespan^16–21^, alternate charge–discharge profiles without any change of current density during whole cycling process have often been used as training datasets. By contrast, herein, we considered random changes in current values during each cycle and predicted the transient process of output voltage using the LSTM technique^22–24^. If such an LSTM model can be constructed, the voltage changes (output) can be calculated for any given current value (input), potentially serving as an input–output device (battery emulator) for prototypes. Generally, during the early stages of research, it is difficult to prepare multiple large-scale prototype batteries for newly proposed materials. However, if an emulator exists, various operational tests for devices using large-scale batteries can be conducted using limited laboratory data. This is expected to significantly reduce the economic and time costs in research and development, from materials to device development. We reported on a battery emulator using the LSTM technique, focusing on its prediction accuracy with respect to the choice of descriptors and the necessary data volume for training.

## Methods

In this study, we prepared all time–current–voltage (*t*–*I*–*V*) datasets by performing two types of galvanostatic charge–discharge tests for LIBs. The first dataset is a simulation dataset generated by the Dualfoil model, a charge–discharge simulator developed by Albertus and Newman^25^. In detail, the simulator considers a set of six coupled differential equations: (1 and 2) a material balance on the electrolyte and the solid electrode, (3 and 4) Ohm’s law in the liquid and solid phases, (5) a current balance between the solid and liquid phases, and (6) Butler–Volmer kinetics^26^. We set Li_x_CoO_2_ as the positive electrode and Li metal as the negative electrode material, and the electrolyte considered was LiTFSI-containing polyethylene oxide at 85 ℃. The material parameter sets used were referred to the initial settings in Dualfoil software (Dualfoil ver 5)^27^. Galvanostatic charging and discharging were performed by randomly changing the current density and duration from the initial charge state of Li_0.5_CoO_2_ to the discharge state of LiCoO_2_ to obtain the *t*–*I*–*V* profile. The cutoff potentials were set to an upper limit of 4.7 V and a lower limit of 2.0 V, and the process was terminated if the cutoff potential was reached before the set time. The current density was varied between − 2.0 and 2.0 A m^−2^, where negative or positive values corresponded to charge or discharge, respectively. The duration of the galvanostatic charge–discharge was set to random values, but the range of random values for discharging was twice as long as that for charging to proceed with the net reaction toward the discharged state. The discharging time range was set from 0 to 345.195 min and the charging time range was set to half of the discharging time range. A random number selected from this range was used as the input time. During discharge to LiCoO_2_, the current density value changed up to 11 times, with the final process being a discharge to the cutoff potential (random galvanostatic charge–discharge). Examples of input files for the Dualfoil simulation are provided in the Supporting Information. The training/validation datasets consisted of a maximum of 100 current–voltage profiles, whereas the test datasets consisted of 20.

The second dataset was an experimental dataset collected from the charge–discharge measurements of liquid-based LIBs by our team. These batteries consisted of a positive electrode material made of LiNi_0.5_Co_0.2_Mn_0.3_O_2_ (NCM523), a negative electrode material made of artificial graphite, a single-layer PE microporous membrane separator, and an electrolyte made of 1 M of LiPF_6_ in EC:EMC = 1:3 v/v + 2wt% VC. Charge–discharge measurements were conducted over six cycles for each of the nine batteries (cells 01–09). All cells were charged and discharged at 0.1 C to the upper (4.2 V) and lower (3.0 V) cutoffs, respectively, at the first and last (sixth) cycles. The cells showed almost no degradation (degradation within 2% of the original capacity of 17.17 mA h) in both the first and sixth cycles for all nine batteries. The random galvanostatic charge–discharge was performed in the first half of each cycle from the second to fifth cycles, where galvanostatic square wave was set at a current ranging from -17.17 to 17.17 mA (-1.0 to 1.0 C) and a duration time of less than 3600 s for charging and 1800s for discharging, randomly. The total square wave number per cycle was also randomly set from 3 to 15 (the final square wave was always charged to the upper cutoff potential). Positive and negative current values corresponded to charging and discharging, respectively. The range of the random charging time was twice as large as the discharging time to complete transitions from a discharged state to a charged state, as in the case of the Dualfoil simulation. In the second half of each cycle, the cell in the fully charged state was discharged to the lower cutoff potential at 0.1 C. Thus, a total of 36 (4 cycles × 9 cells) *t–I–V* profiles from a fully charged state to a fully discharged state were obtained by random galvanostatic charge/discharge measurements. The training/validation datasets consisted of 30 current/voltage profiles, whereas the test datasets consisted of 8 current/voltage profiles (additional 2 data were used from galvanostatic charge measurements (0.1 C) at the first and sixth cycles).

**Fig. 1:**
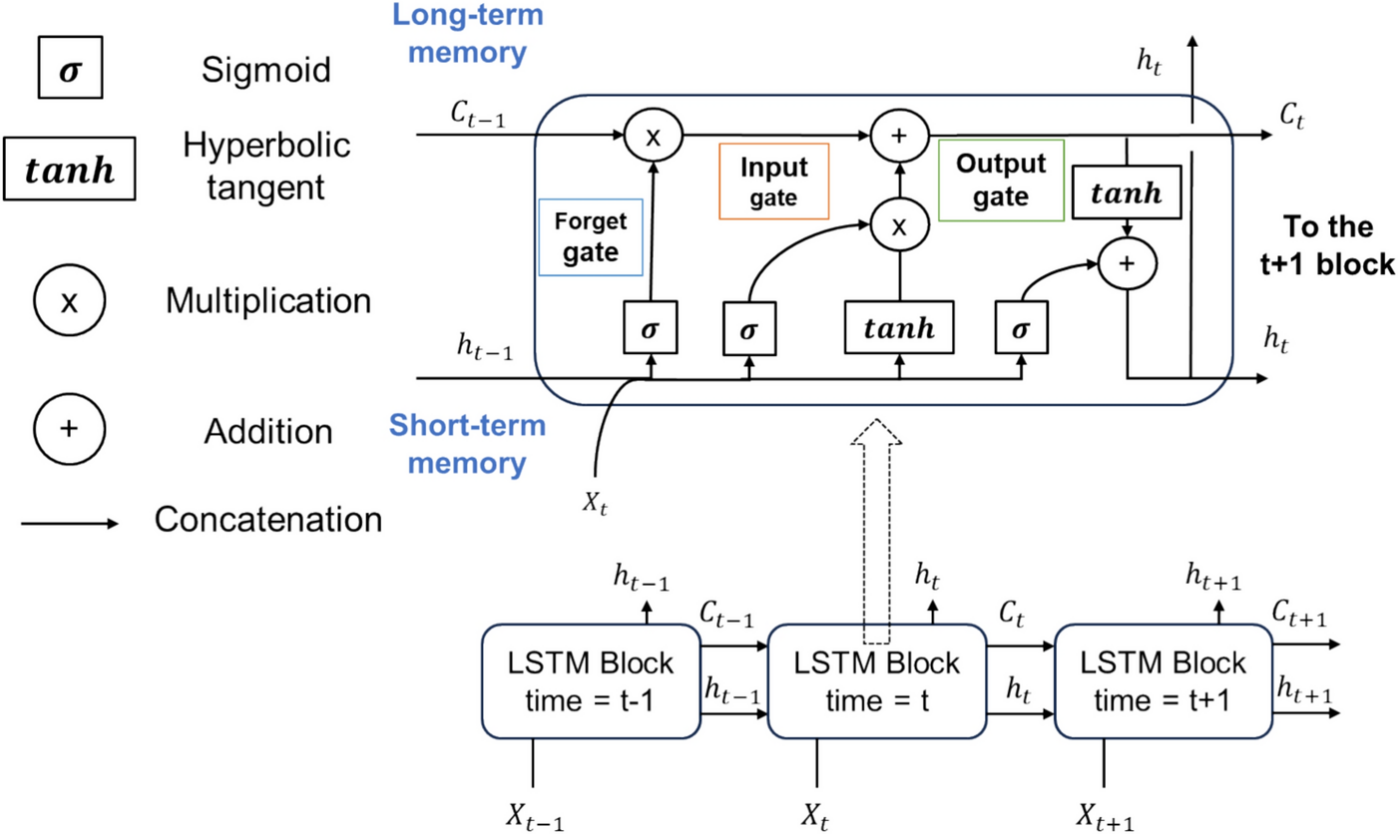
Detailed structure of the long short-term memory (LSTM) block in the hidden layer.

The machine learning model used in this study was LSTM^28^, a deep learning technique for time-series data. This model introduces LSTM blocks into the hidden dimensions to solve the gradient explosion and gradient disappearance problems encountered during computations, unlike a traditional recurrent neural network^29^. Figure 1 shows the LSTM structure used in this study, which consists of a series of LSTM blocks, each of which has inputs (*x*_*t*_) and outputs (*h*_*t*_) at each time step *t*. Of note, *h*_*t*_ is used as the input variable for the LSTM block of the next time step *t* + *1*, which is known as short-term memory. The other output variable *C*_*t*_, which is the input for *t* + *1*, is the long-term memory, as shown in Fig. 1. Each block includes input, output, and forget gates, and is referred to as an LSTM block. Specifically, sigmoid layers and sum-product calculations with a hyperbolic tangent layer were incorporated to determine the memory-forgetting rate. These gates allow for the deletion and addition of information to the cell state, with the LSTM blocks connected continuously according to the hidden dimension parameter. The input gates control when and to what extent new information enters the memory. Forget gates control the degree and timing of information forgetting, allowing the cell state to discriminate between important and superfluous data. For example, noise can be identified using this method. The output gate multiplies the information stored in the cell state with the applied hyperbolic tangent, the information in the hidden state of the previous block, and the input information with the sigmoid function and then transmits it to the next LSTM hidden block. In this study, we used one LSTM layer and fixed the number of hidden dimensions at 30.

In this study, the input variables *x*_*t*_ are the current (current density in the simulation dataset) *I(t)* and *SOC(t)*, where *t* corresponds to time. The SOC of a battery represents the current charge level as a fraction of its total capacity, which is calculated as the sum of the current and time from a fully discharged state. The output variable *h*_*t*_ is the voltage *V(t)*, which is also used as the input for the LSTM block at *t* + *1*, as shown in Fig. 1. A total of 30 sequential datasets of *I(t)*, *SoC(t)*, and *V(t)* were input for the long-term memory *Ct*.

In the LSTM block at *t* + *1*, the voltage value *V(t* + *1)* is predicted based on the user-specified current (*I(t* + *1)*) and SOC values (*SOC(t* + *1)*), which are considered as inputs and short-term memory, *h*_*t*_ = *V(t)*, and *C*_*t*_ from one prior block. Using the predicted voltage *V(t* + *1)*, it is possible to repeatedly predict the voltage values with the user-specified current and SOC inputs. In this study, we assumed situations in which the current value changes abruptly due to arbitrary user operations. Hence, the prediction step length is fixed at one step; otherwise, it would be impossible to make the predicted voltage value follow significant current value changes.

Training data were learned with mini-batch learning using a maximum of 100 or 30 random charge–discharge profiles for the simulation or experimental dataset, respectively. Our hyperparameters included the hidden dimension, number of LSTM layers, maximum number of epochs, learning rate, and mini-batch size. Because the hidden dimension and number of LSTM layers were fixed at 30 and 1, respectively, we performed grid searching for learning rate and mini-batch size. The number of maximum epochs was set to 5000 and 15,000 for simulation and experimental datasets, respectively, and an early stopping technique was used. The best hyperparameters, learning rate, batch size, and epochs for the LSTM were determined using K-fold cross-validation (K up to 10) of the training data. We found no significant differences among selected hyperparameters (The maximum RMSE was 0.07 eV when using experimental data.), and the best fitting results were subsequently used. A comparison of prediction error differences during the validation process using grid search is provided in the supplementary data.

## Results and discussion

### LSTM for the simulation-driven charge–discharge profile

**Fig. 2:**
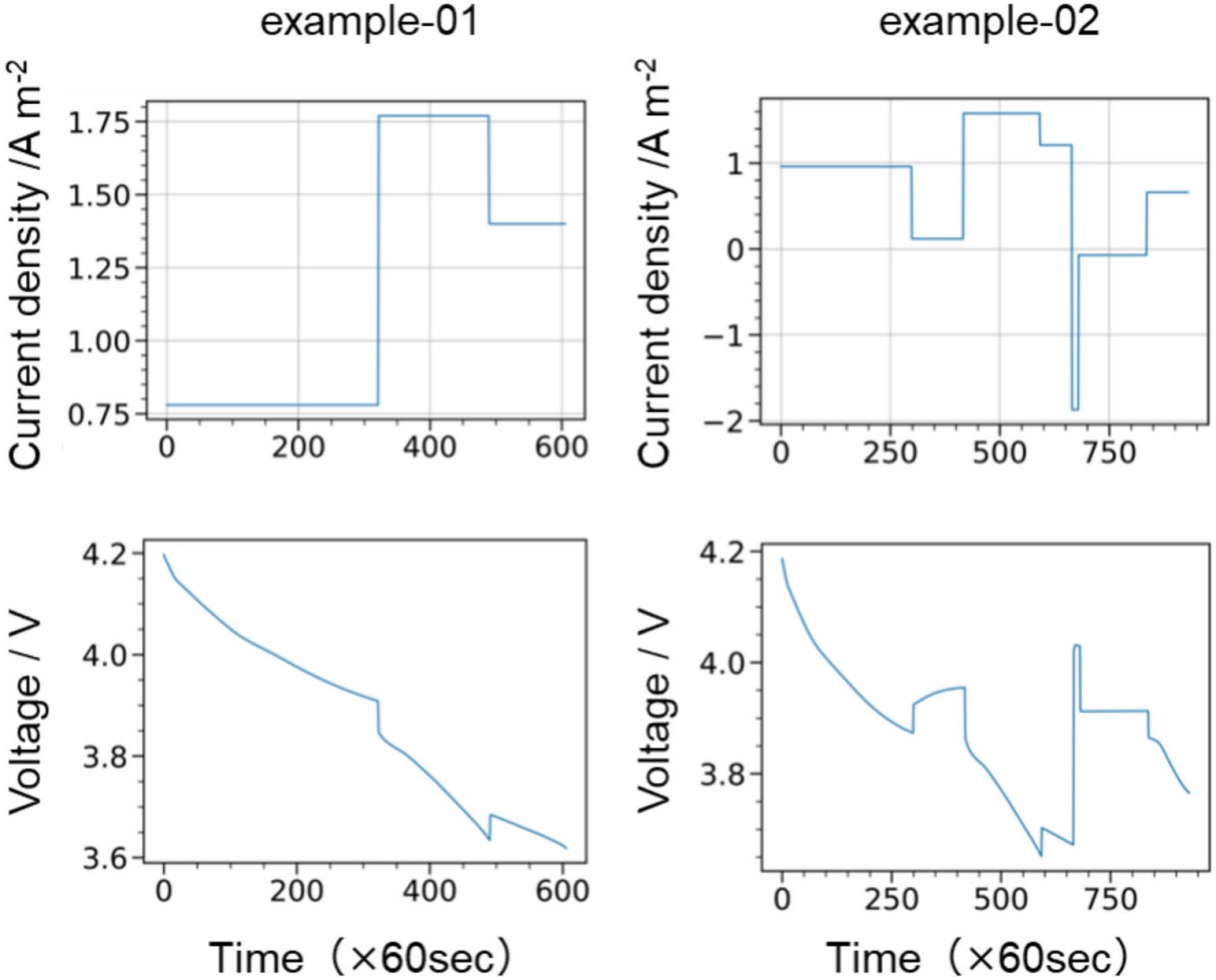
Representative charge–discharge dataset obtained from Dualfoil, current density square wave (top row), and the corresponding potential profile behaviour (bottom row).

**Fig. 3:**
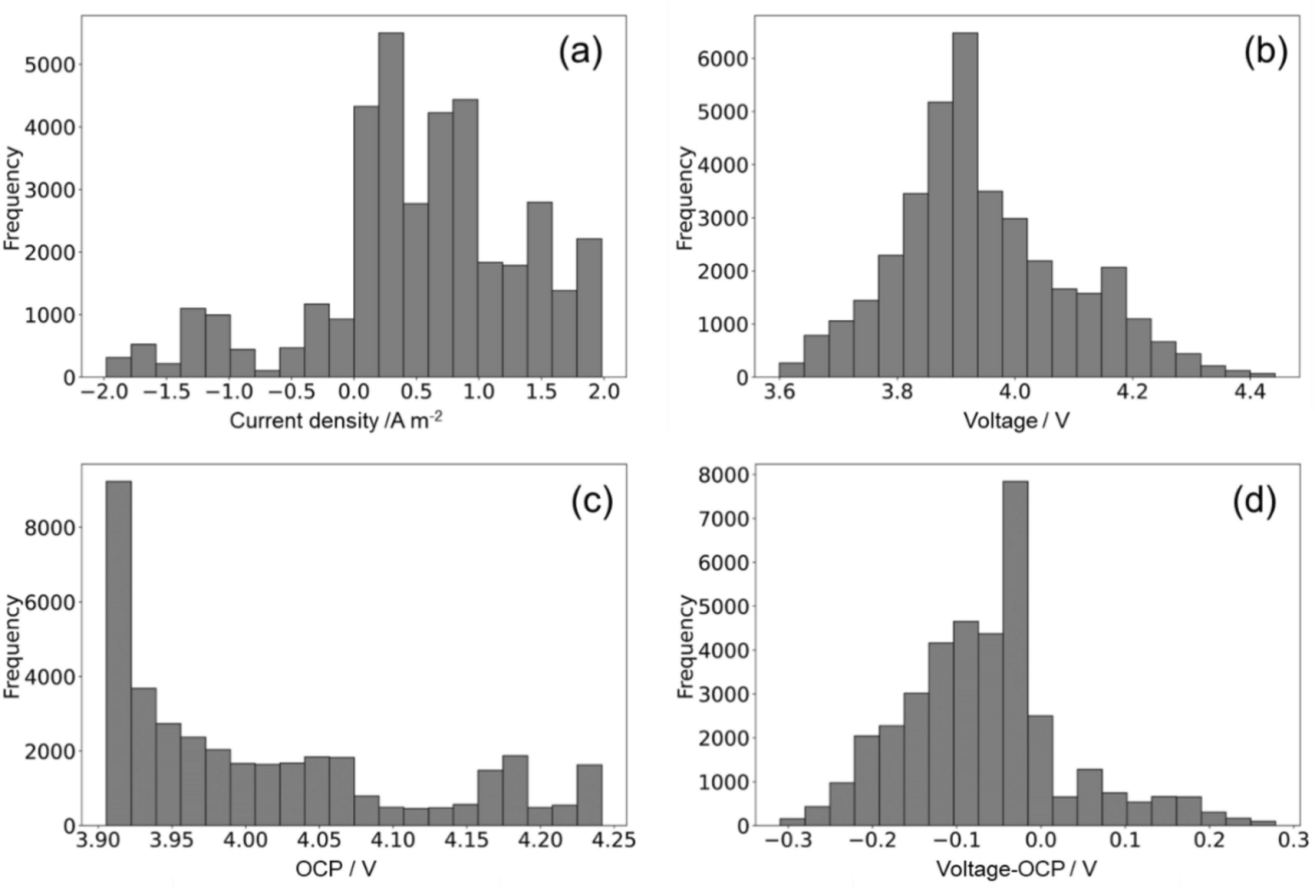
Histograms of (**a**) current density values, (**b**) voltage values, (**c**) voltage values and open circuit potential (OCP), and (**d**) OCP reference time for a total of 30 random charge–discharge datasets.

Figure 2 shows a representative charge–discharge dataset obtained using the Dualfoil simulation. Figure 3a–d show the histograms of 30 randomly obtained charge–discharge curves used for the training datasets, focusing on the current density value, voltage value, open circuit potential (OCP) obtained during the simulation, and the difference between the OCP and the voltage value obtained at each step break. The total training/validation *t*–*I*–*V* dataset consisted of 37,532 steps (simulation in min.). Figure 3a shows that the large distribution on the discharge side is apparent owing to the Dualfoil simulation settings, where the range of the discharge time was set to be twice as large as that of the charge time to ensure that the reactions proceeded in the discharge direction. Figure 3b shows that the voltage values in the dataset generally follow a normal distribution, with ample data collected near the median of the voltage distribution during charge–discharge but fewer data points at the end of charging and discharging. Figure 3d shows that the voltage difference from the OCP (overvoltage) is within ± 0.3 (V), with fewer data points as the voltage difference increased.

**Fig. 4:**
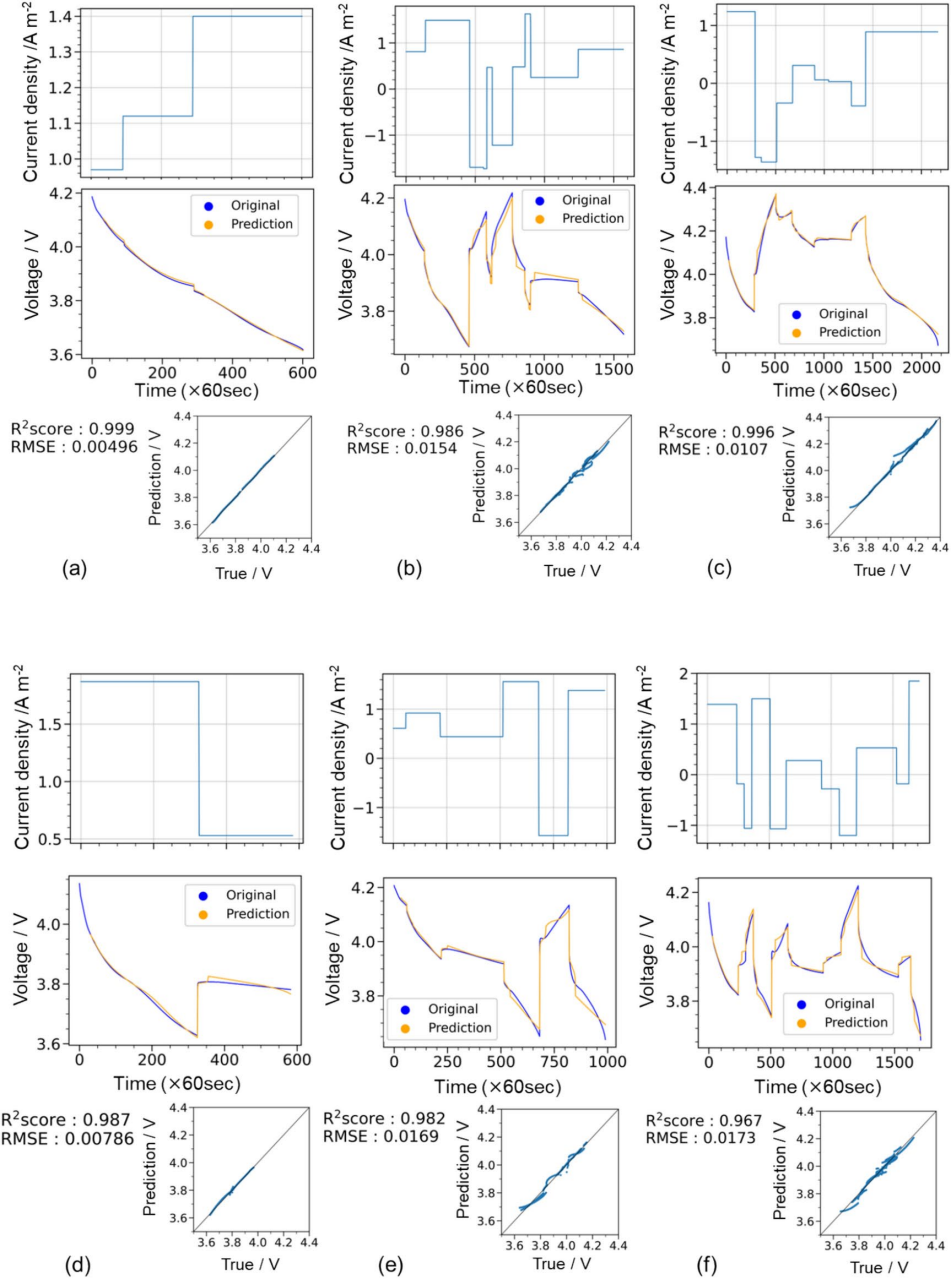

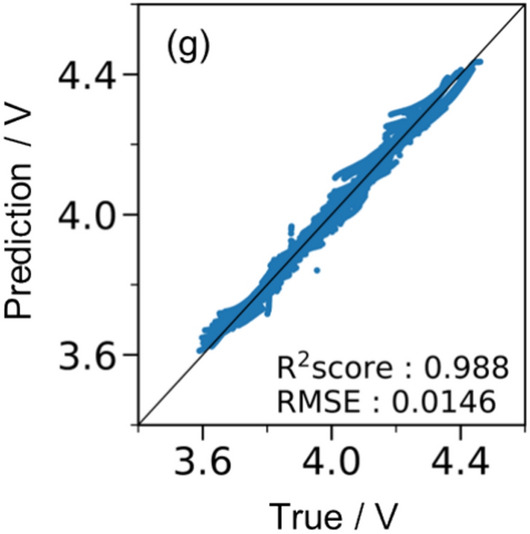
(**a**–**f**) Predictions for training data that also confirm the accuracy of the training model and an example of a test prediction. Charge–discharge schedule set in Dualfoil (upper row) and the corresponding voltage behaviour (blue line; middle row); voltage behaviour prediction when 30 points are used as initial points (orange line; lower part). (**g**) Diagnostic plots summarising the prediction results for 20 test data.

Figure 4a–f show the *t*–*I*–*V* relationship, where the top and bottom correspond to random galvanostatic square-wave inputs and voltage outputs, respectively. The voltage profiles were obtained through (1) LSTM prediction trained using 30 *t*–*I*–*V* profiles (orange) and (2) the Dualfoil simulation (blue). Figure 4a–c show the examples of prediction results for the training data, and Fig. 4d–f show the examples of the test data. Figure 4(g) shows the diagnostic plots of LSTM-predicted and Dualfoil-simulated voltages for the 20 test datasets. In this study, the coefficient of determination^30^ (R^2^ score) and the RMSE^31^ expressed in Eqs. (1) and (2), respectively, were used as measures of prediction accuracy.

1 $${R}^{2}=1-\frac{\sum_{i=1}^{n}{\left({y}_{i}-\widehat{{y}_{i}}\right)}^{2}}{\sum_{i=1}^{n}{\left({y}_{i}-\overline{y }\right)}^{2}}$$

2 $$RMSE=\sqrt{\frac{1}{n}\sum_{i=1}^{n}{\left({y}_{i}-\widehat{{y}_{i}}\right)}^{2}}$$

The total number of steps in which the voltage was predicted is denoted by *n*, the Dualfoil-simulated voltage is denoted by ${y}_{i}$, and the average of ${y}_{i}$ is $\overline{y }$. The LSTM-predicted voltage is denoted by $\widehat{{y}_{i}}$.

The predictions for the training data illustrated in Fig. 4a–c show that, in all three cases, the behaviour was predicted very accurately, with R^2^ scores exceeding 0.99. We compared the *t*–*I*–*V* profile between cases with fewer galvanostatic square waves (< 5) and those with more square waves (≥ 5) and that the former showed a good match (Fig. 4a), whereas the latter exhibited slight discrepancies in voltage (Fig. 4b and c). These discrepancies are likely due to the large discontinuous changes in the current density values. In particular, the discrepancy in voltage profile changes were visible 30 steps after a discontinuous change in the current density; therefore, the insufficient prediction is attributed to the inability to access the data at the discontinuous change point because the present setting refers to 30-step-past data. An improvement is expected by extending the reference (past) data inputs, although our graphical processing unit environment limits the further extension of past data. LSTM predictions shown in Fig. 4d–f for the test data indicate a slight decrease in prediction accuracy compared to LSTM predictions for the training data. Nevertheless, LSTM predictions showed good accordance with the Dualfoil-simulated behaviour for the three test cases, and the R^2^ score exceeded 0.98 in the diagnostic plot of Fig. 4g for all 20 test datasets. This indicates the ability to accurately estimate the charge–discharge voltages for arbitrary *t–I* inputs.

**Fig. 5:**
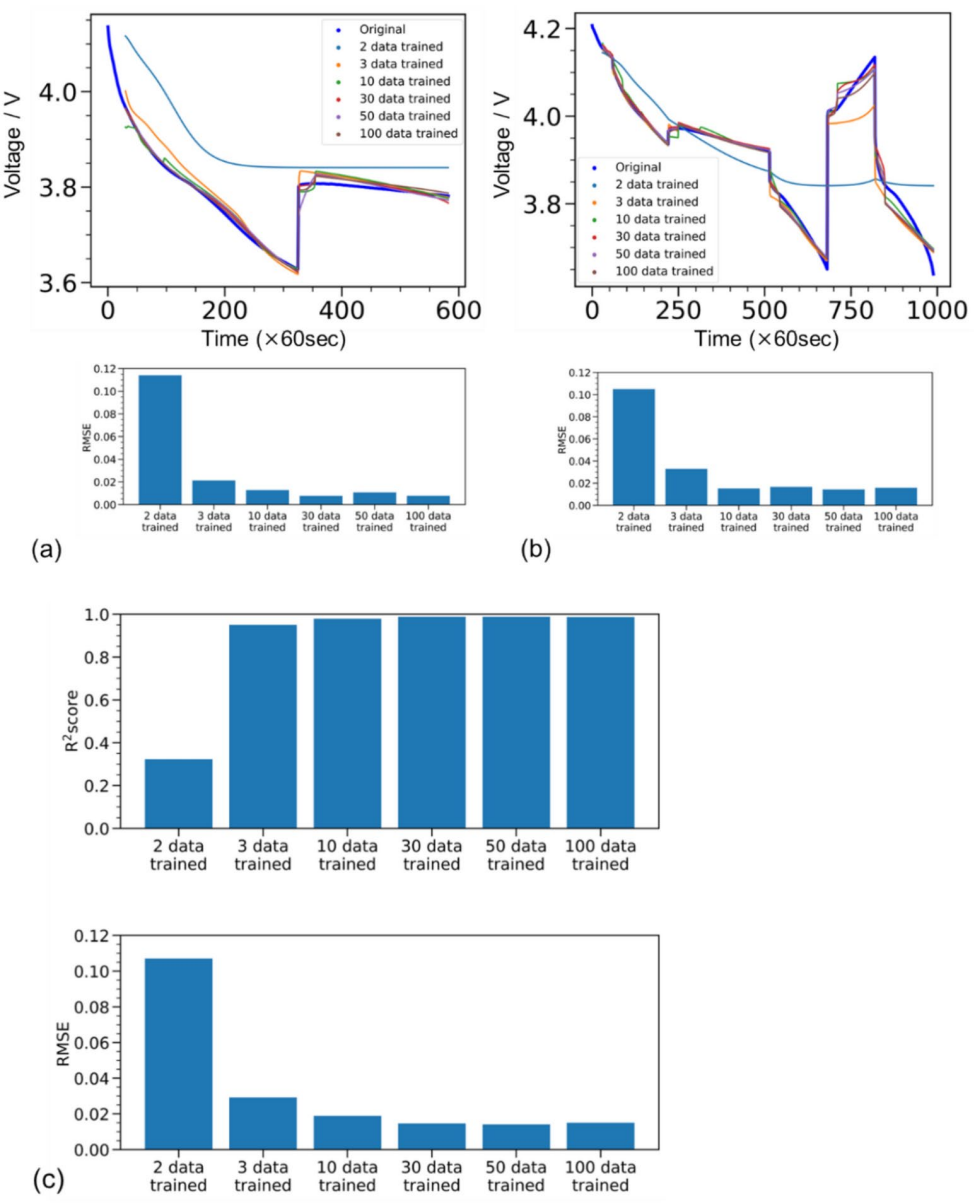
Example comparison of the prediction accuracy of test data for different numbers of training data used to build the prediction model. (**a**) Prediction results and root mean square (RMSE) trends for test data with a relatively simple voltage behaviour. (**b**) Trends in prediction accuracy and RMSE values for test data with a complex voltage behaviour. (**c**) Trends in R^2^ scores and RMSE values for 20 test datasets.

The relationship between the amount of training data used to build the LSTM prediction model and the prediction accuracy of the test data was investigated. Figure 5a and b show a comparison between the LSTM-predicted and Dualfoil-simulated voltage profiles for (a) fewer and (b) more galvanostatic square waves, respectively. In both cases, a large deviation between the predicted and simulated voltage profiles was observed when the number of training data points was two. By contrast, the deviation was largely improved when more than three galvanostatic charge–discharge data were trained in both cases (a) and (b). The RMSE was slightly higher in the case shown in Fig. 5b because of the complex voltage profile; however, the difference was minor. Figure 5c shows the R^2^ and RMSE values for all 20 test datasets, showing a trend similar to that in Fig. 5a and b. The RMSE was largely decreased owing to the use of more than three training datasets, whereas it slightly decreased when the number of training datasets was increased from 3 to 100. Thus, collecting less than 10 training data points may be sufficient to reproduce the *t*–*I*–*V* relationship, which would be helpful and attractive because the number of prototype cells available is often limited.

**Fig. 6:**
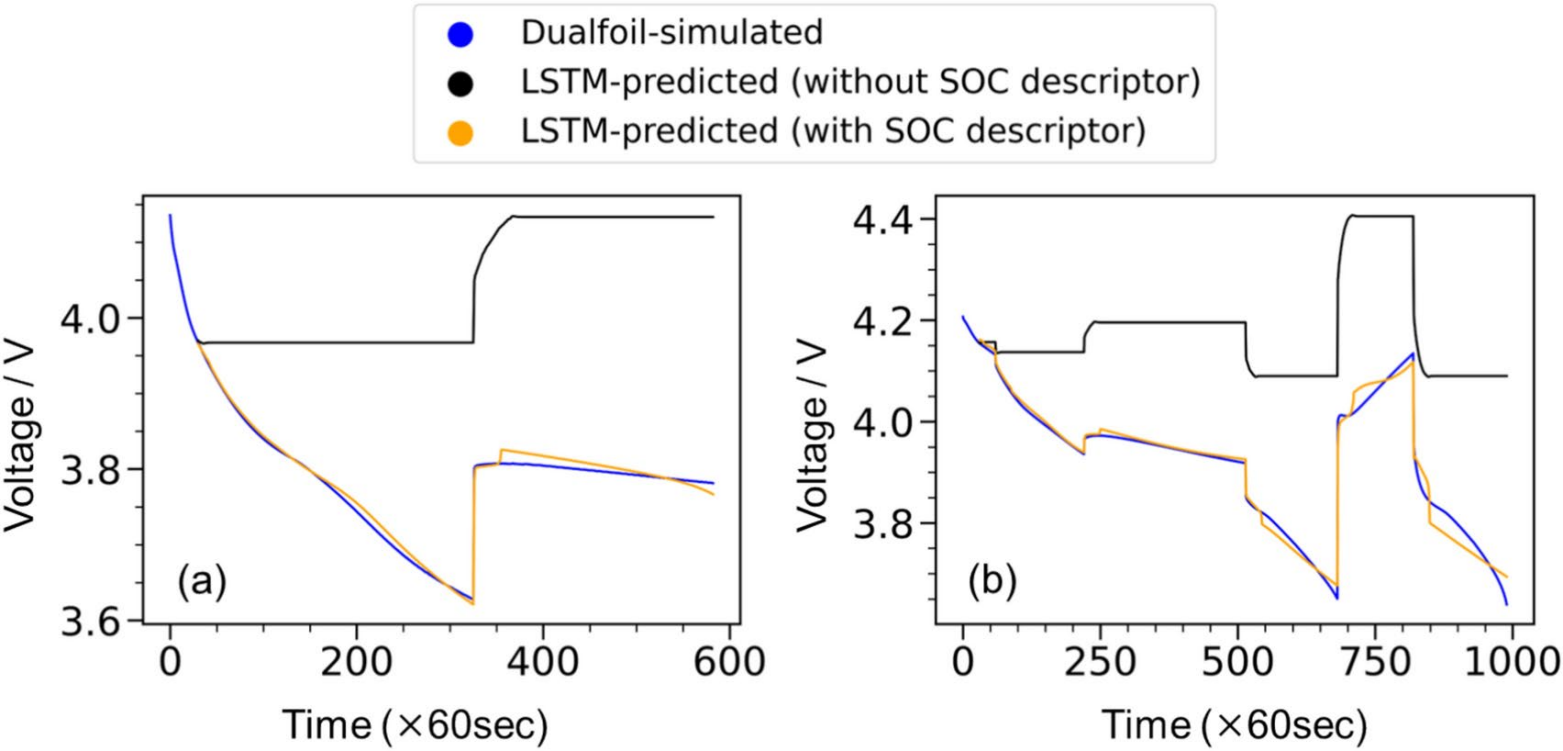
Comparison of machine learning accuracy with and without state of charge (SOC) descriptors. (**a**) Test data with a relatively simple voltage behaviour and (**b**) test data with a complex voltage behaviour.

We also investigated the prediction accuracy against the choice of descriptors used. Until now, we included the SOC descriptor; however, the SOC is not an independent parameter but a derivative of *t*–*I* parameters because there have been research reports on the prediction of battery degradation and cycle estimation using SOC information^32–34^. Figure 6 shows the LSTM-predicted voltage profile with and without SOC descriptors. The inclusion of the SOC descriptor is crucial for prediction accuracy. We inferred that the importance of the SOC descriptor is related to the OCP. Indeed, the LSTM-predicted voltage profile without the SOC descriptor was flat under a constant current charge–discharge region, as shown in Fig. 6a and b. Hence, descriptor I was mainly used to reproduce the overpotential of the cell.

In addition to the LSTM, we conducted comparative studies on other deep learning models capable of time-series learning (i.e., RNN^35^ and GRU^36^). Similar to the time-series analysis using LSTM (Fig. 5), we used three charge–discharge profiles from the *t–I–V* profiles generated by the Dualfoil simulation as training data. We then evaluated the reproducibility for 20 test samples using RMSE and R^2^ of the diagnostic plots. As shown in Supporting Fig. S1, the RNN model exhibited relatively poorer fit in the diagnostic plots than the LSTM, demonstrating the superiority of LSTM in maintaining long-term memory. By contrast, the GRU results were nearly identical in accuracy to those of the LSTM model. These findings suggest that the GRU model may also be a viable option for achieving the objectives of this study.

### LSTM for the experimental charge–discharge profile

**Fig. 7:**
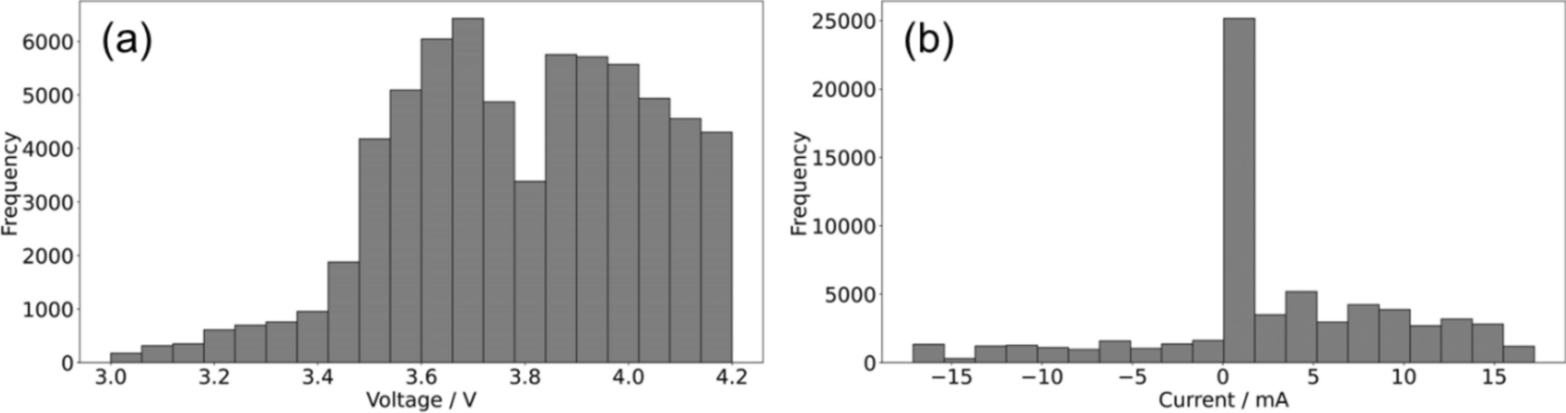
Histogram of time with reference to (**a**) current values and (**b**) voltage values for a total of 30 experimental charge and discharge cycle datasets, excluding the fully discharge step in the final step.

Figure 7 shows the statistics of the measured* t*–*I*–*V* profiles for 30 random experimental datasets. The voltage value distribution is skewed to the larger side in Fig. 7a because the range of the randomly generated duration time for charging the square wave is twice that for discharging. Figure 7b shows a histogram of current values, showing a skew toward the positive side. The total learning amount for the 30 datasets was 165,859 steps, corresponding to 27,643 min (~ 460 h/30 cells).

**Fig. 8:**
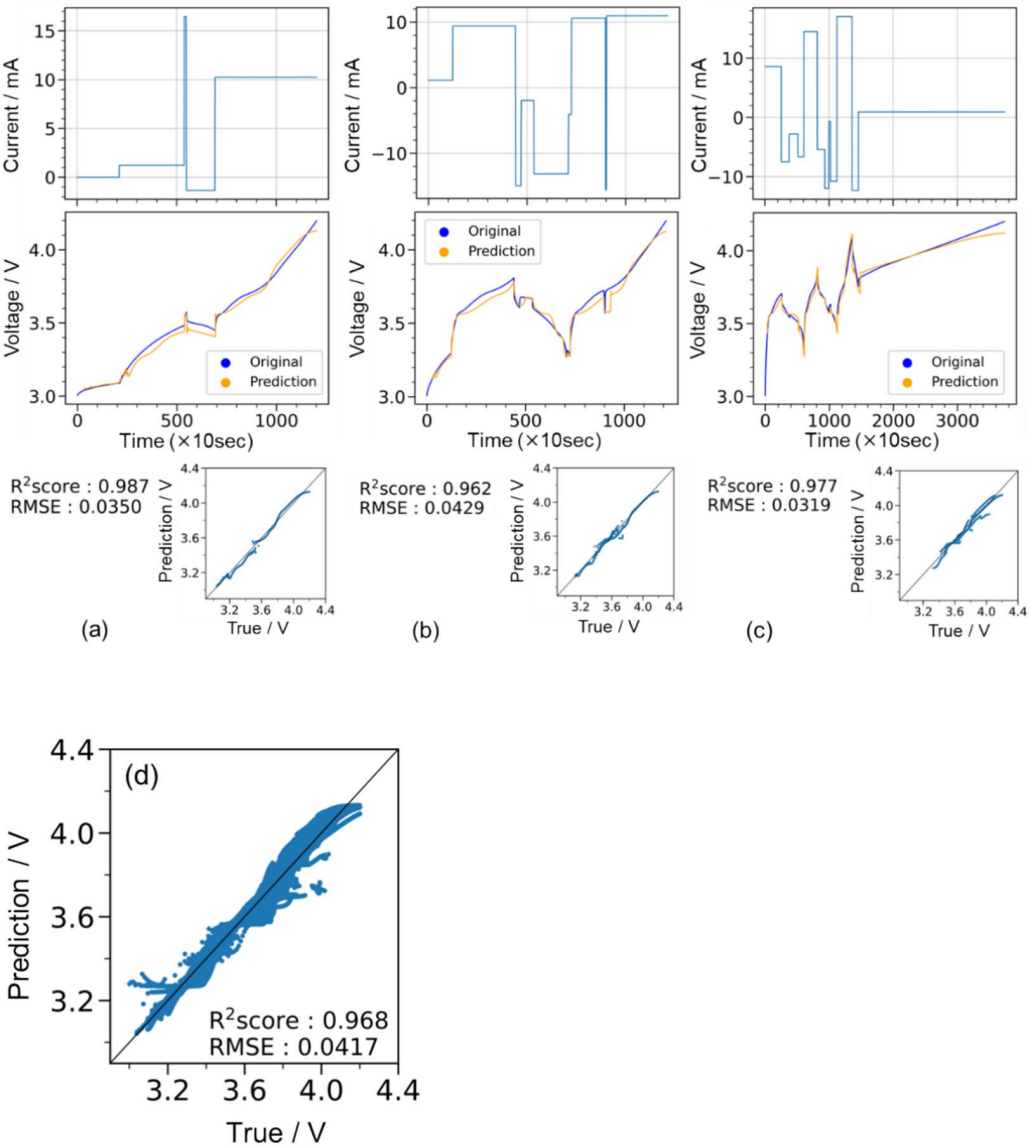

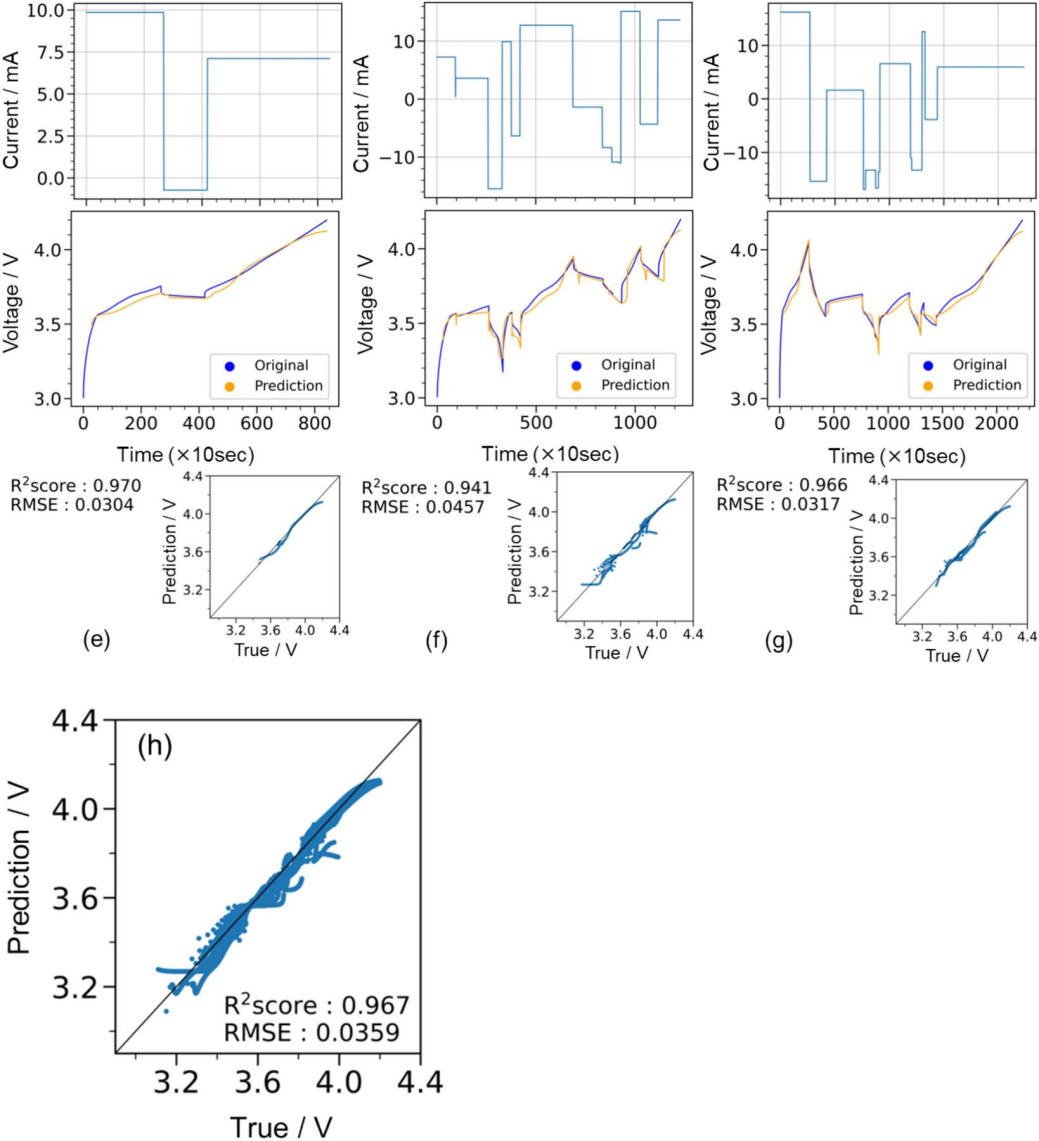
(**a**–**g**) Predictions for training data that also confirm the learning model and examples of test predictions. Charging and discharging schedule (upper row) and the corresponding voltage behaviour (blue line; middle row) set up in an experiment on a real battery, and voltage behaviour prediction (orange line; lower row) when 30 data points are used as initial points. (**d**) and (**h**) Diagnostic plots summarising the prediction results for all 30 training data and eight test data.

**Fig. 9:**
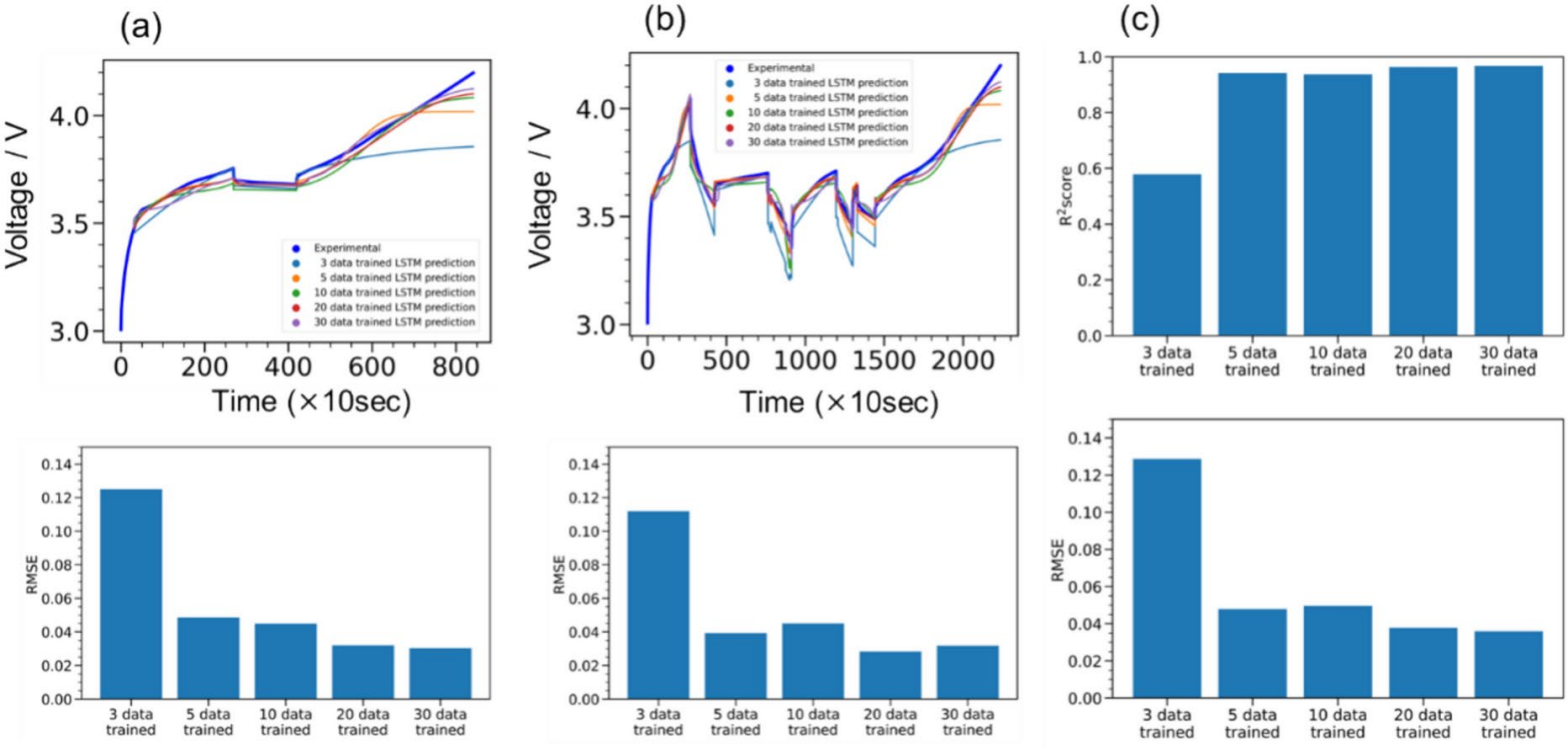
(**a**,**b**) Top of each panel shows an example of LSTM-predicted and experimentally observed voltage profiles in test datasets, where the number of training datasets varied from 3 to 30. Bottom bar graphs show the corresponding RMSE values, which changed with the number of training datasets. (**c**) Top and bottom bar graphs show R^2^ scores and RMSE values, respectively, for all eight test datasets, with the number of training datasets ranging from 3 to 30.

In the prediction of the training data shown in Fig. 8a–c, the R^2^ score exceeded 0.96 in all three cases, and the model was able to follow and make predictions even in areas where the applied current switched in detail, indicating that the prediction model for the training data of the experimental potential profile was successfully constructed, similar to that with the simulation data. The prediction results for all 30 training datasets shown in Fig. 8d, again using the best-constructed model, also indicated that the prediction performance of the model for the training data was accurate. The R^2^ scores were all above 0.94 when considering the three test examples shown in Fig. 8e–g, indicating good agreement between the LSTM-predicted and experimental voltage profiles. Figure 8h shows the diagnostic plots of LSTM-predicted and measured voltages for all eight test data, where the R^2^ score exceeded 0.96, confirming the high prediction and generalisation performance. We also investigated the RMSE as a function of SOC (SOC = 25, 50, 75, and 100%) and confirmed that there was no significant increase in the prediction error, even in the high SOC region (far from the input *t*–*I*–*V* profile), as listed in Supporting Table S1. Figure 9 shows the evaluation of the reproduction performance of the voltage profile based on the size of the training data, like that shown in Fig. 5. Figure 9a and b show the voltage profile reproduction examples in the test data when the training data size varied (3, 5, 10, 20, and 30). The results indicated that, even with only three datasets, the general shape of the voltage profile could be approximately traced. The RMSE value significantly decreased when the training data were increased from three to five. Figure 9c presents the results using all eight test data. Consistent with the trends in (a) and (b), the RMSE (R^2^) values significantly decreased (increased) from three to five dataset inputs for training. When the training dataset was increased from 5 to 10, RMSE (R^2^) values slightly increased (decreased), suggesting that, with the use of very few training data points, performance may slightly vary depending on the selection of the training/test dataset. Accordingly, the experimental results showed that charge–discharge profiles could also be emulated as demonstrated in the LSTM prediction using Dualfoil simulation datasets.

## Conclusion

In this study, we focused on testing the performance of LIBs used in EV development to reduce the economic costs and time associated with the fabrication of large-scale automotive prototype batteries by building a data-driven battery emulator. The machine learning model used was LSTM, a time-series deep learning technique that has been reported to be useful for predicting the lifetime and cycle degradation of LIBs. Two datasets were used: a simulation dataset collected from Dualfoil and an experimental dataset collected from liquid-based LIBs. We aimed to reproduce and analyse the charging and discharging behaviours by predicting the potential profile for an arbitrary charging and discharging schedule. Consequently, test data predictions for both the simulation and experimental datasets achieved a high prediction accuracy of charge–discharge behaviour, with R^2^ scores of 0.98 and 0.97 for each test data example, respectively. The training model was successfully built to achieve a highly accurate fit, and the importance of SOC descriptors was also verified. In addition, the relationship between the training data size and the robustness of the model was investigated in the abovementioned test data prediction, confirming that the model performance was high even with a small number of training data (approximately 10 charge/discharge data) and that the model performance improved as the number of training data increased.

The research conducted in this study is expected to be beneficial in scenarios where large prototype batteries are required. By constructing a data-driven emulator, it is possible to link the data obtained from small prototype batteries at the research stage to large prototype batteries required for developing devices. This approach is anticipated to be useful for shortening the research and development periods. Furthermore, we can repurpose many of the deep learning methods developed thus far. For instance, by referencing a one optimised to reproduce the charge–discharge behaviour of a certain prototype battery, high-accuracy predictions become possible through transfer learning, even with very limited data for the new prototype battery. Moreover, if sufficient cycle degradation data can be prepared, it would become possible to perform battery life diagnostics for prototype materials using complex *t–I–V* histories.

## Supplementary Information

Supplementary Information.

## Data Availability

Scripts and charge/discharge data by simulations and experiments are available in Figshare: doi: 10.6084/m9.figshare.26362066.
