# Supplementary material for: Deep learning based emulator for predicting voltage behaviour in lithium ion batteries
Source: Sci Rep. 2024 Nov 21;14:28905. doi: 10.1038/s41598-024-80371-9 (PMC11582594; doi:10.1038/s41598-024-80371-9)
Supplement: Supplementary file 1 — Supplementary Information. [file 41598_2024_80371_MOESM1_ESM.docx]

Supplementary Information for

**Development of a battery emulator using deep learning model to predict the charge–discharge voltage profile of lithium-ion batteries**

Kanato Oka^1^, Naoto Tanibata^1^, Hayami Takeda^1^, Masanobu Nakayama^1,*^, Syuto Noguchi^2^, Masayuki Karasuyama^2^, Yoshiya Fujiwara^3^ and Takuhiro Miyuki^3^

^1^Department of Advanced Ceramics, Nagoya Institute of Technology, Gokiso, Showa-ku, Nagoya, Aichi 466-8555, Japan

^2^Department of Computer Science, Nagoya Institute of Technology, Gokiso, Showa-ku, Nagoya, Aichi 466-8555, Japan

^3^Consortium for Lithium Ion Battery Technology and Evaluation Centre (LIBTEC), 1-8-31 Midorigaoka, Ikeda, Osaka 563-8577, Japan

***Corresponding author:** Masanobu Nakayama

Email ID: masanobu@nitech.ac.jp

Table S1: Root mean square error (RMSE) for voltage prediction according to state of charge (SOC) status for eight experimental test data

| SOC /% | 25 | 50 | 75 | 100 |
| --- | --- | --- | --- | --- |
| RMSE for eight experimental test data | 0.049 | 0.042 | 0.024 | 0.075 |


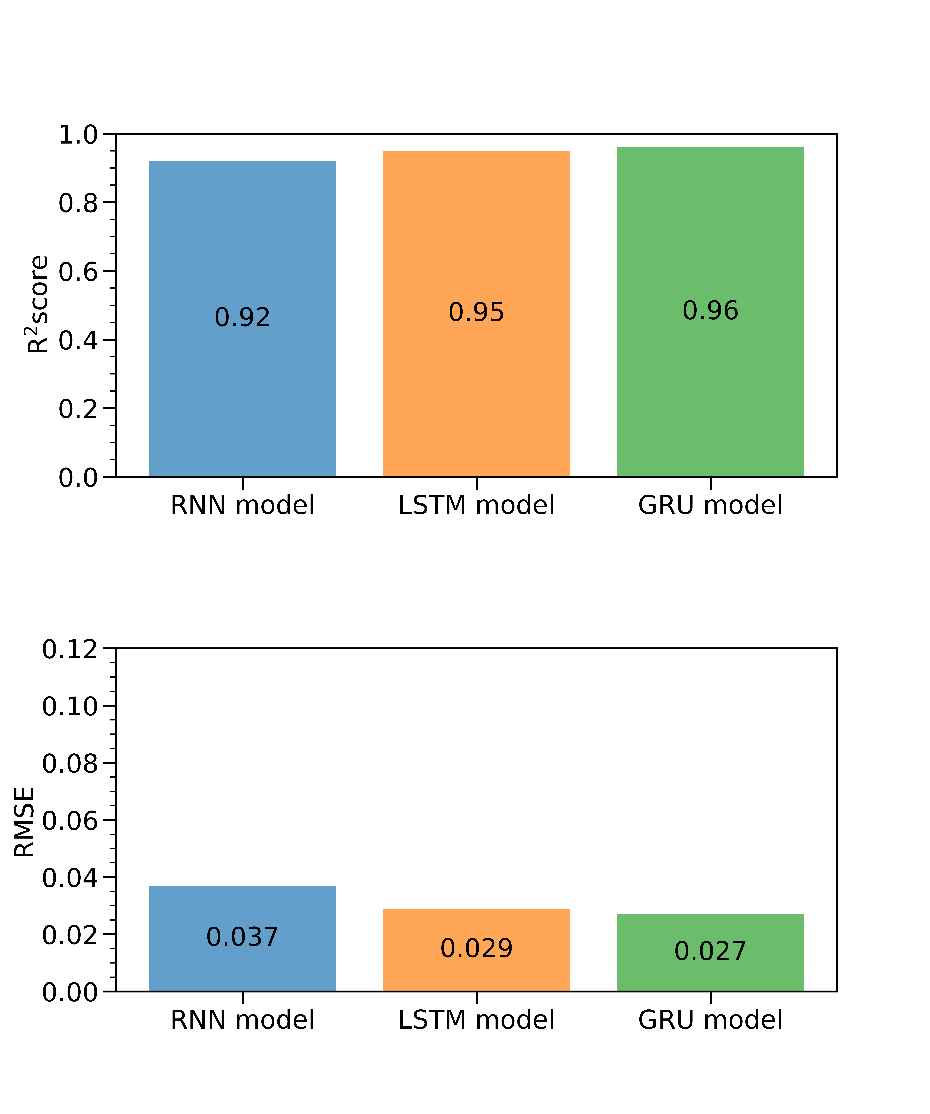


(b)


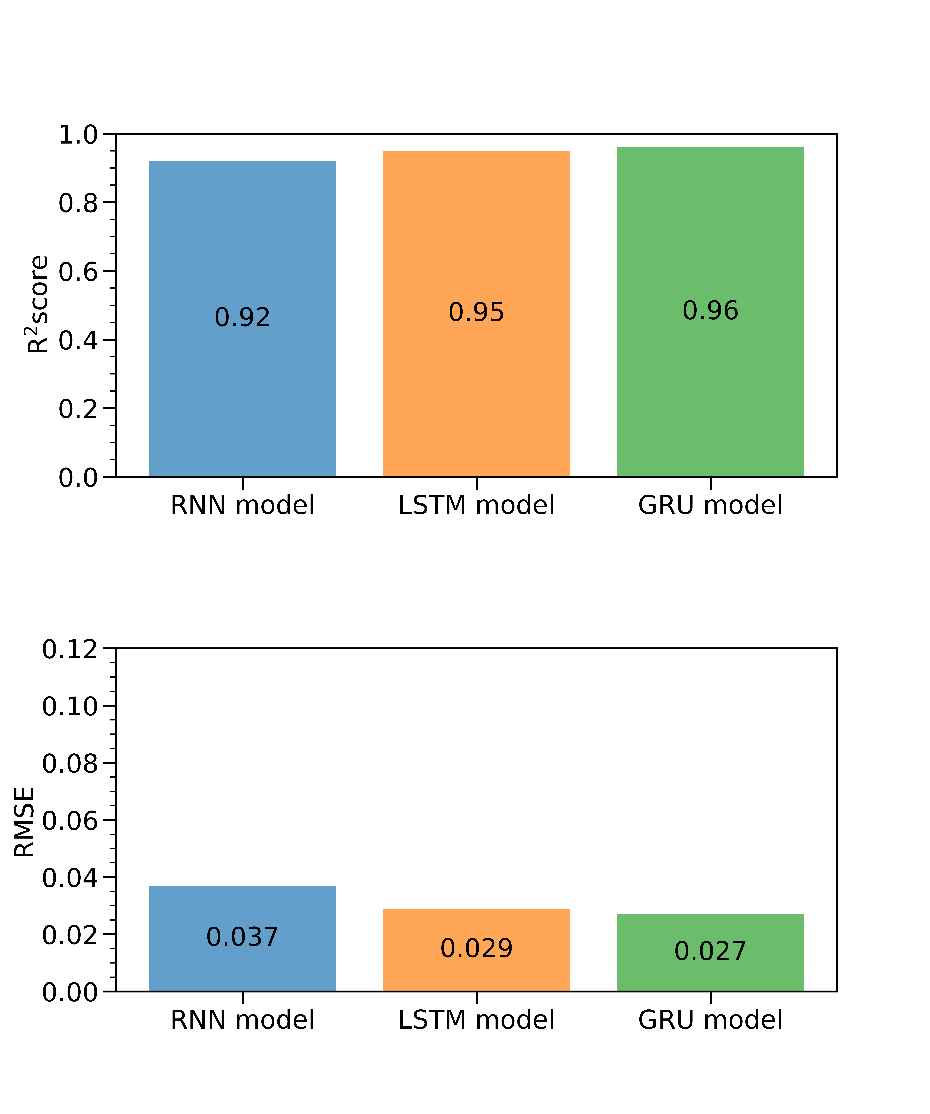


**Fig. S1.** Comparison of (a) R² score and (b) root mean square error (RMSE) for eight test data prediction accuracy using the long short-term memory (LSTM) model with minimal training data and other time series deep learning models (i.e., recurrent neural network (RNN) and gated recurrent unit (GRU)).
